# Total staphylococci as performance surrogate for greywater treatment

**DOI:** 10.1007/s11356-017-9050-1

**Published:** 2017-05-01

**Authors:** David C. Shoults, Nicholas J. Ashbolt

**Affiliations:** School of Public Health, University of Alberta, Room 3-57, South Academic Building, Edmonton, AB T6E 2G7 Canada

**Keywords:** Greywater, Graywater, *Staphylococcus*, Staphylococci, Water treatment, Indicator organisms, Surrogates, UV disinfection, Collimated beam

## Abstract

Faecal indicator bacteria (FIB) are commonly used as water quality indicators; implying faecal contamination and therefore the potential presence of pathogenic enteric bacteria, viruses, and protozoa. Hence in wastewater treatment, the most commonly used treatment process measures (surrogates) are total coliforms, faecal coliforms, *Escherichia coli* (*E.* c*oli*), and enterococci. However, greywater potentially contains skin pathogens unrelated to faecal load, and *E. coli* and other FIB may grow within greywater unrelated to pathogens. Overall, FIB occurs at fluctuating and relatively low concentrations compared to other endogenous greywater bacteria affecting their ability as surrogates for pathogen reduction. Therefore, unlike municipal sewage, FIB provides a very limited and unreliable log-reduction surrogate measure for on-site greywater treatment systems. Based on our recent metagenomic study of laundry greywater, skin-associated bacteria such as *Staphylococcus*, *Corynebacterium,* and *Propionibacterium* spp. dominate and may result in more consistent treatment surrogates than traditional FIB. Here, we investigated various *Staphylococcus* spp. as potential surrogates to reliably assay over 4-log_10_ reduction by the final-stage UV disinfection step commonly used for on-site greywater reuse, and compare them to various FIB/phage surrogates. A collimated UV beam was used to determine the efficacy of UV inactivation (255, 265 and 285 nm) against *E. coli*, *Enterococcus faecalis*, *E. faecium*, *E. casseliflavus*, *Staphylococcus aureus,* and *S. epidermidis*. *Staphylococcus* spp. was estimated by combining the bi-linear dose-response curves for *S. aureus* and *S. epidermidis* and was shown to be less resistant to UV irradiation than the other surrogates examined. Hence, a relative inactivation credit is suggested; whereas, the doses required to achieve a 4 and 5-log_10_ reduction of *Staphylococcus* spp. (13.0 and 20.9 mJ cm^−2^, respectively) were used to determine the relative inactivation of the other microorganisms investigated. The doses required to achieve a 4 and 5-log_10_ reduction of *Staphylococcus* spp. resulted in a log_10_ reduction of 1.4 and 4.1 for *E. coli*, 0.8 and 2.8 for *E. faecalis*, 0.8 and 3.6 for *E. casseliflavus* and 0.8 and 1.2 for MS2 coliphage, respectively. Given the concentration difference of *Staphylococcus* spp. and FIB (3 to 5-log_10_ higher), we propose the use of *Staphylococcus* spp. as a novel endogenous performance surrogate to demonstrate greywater treatment performance given its relatively high and consistent concentration and therefore ability to demonstrate over 5-log_10_ reductions.

## Introduction

Available freshwater is an increasingly scarce commodity for many rapidly urbanizing regions, even within developed countries (World Health Organization 2016). However, increasing population growth in relatively water-scarce regions along with an increase in personal water consumption have greatly contributed to the urban water deficit faced around the world (Schiermeier 2014). There is also a greater awareness in the water-energy nexus (Sathe 2013), and that most household water use does not need to be treated to drinking water quality. In particular, many circumpolar communities lack adequate quantities of water for potable and non-potable purposes (Daley et al. 2014; Hennessy and Bressler 2016). Hence, one option to provide more sustainable water services is to utilize treated greywater (Schoen et al. 2014). Greywater (graywater), which can be defined as domestic household wastewater without input from the toilet (Ottosson 2003), is a valuable commodity which should be utilized to reduce water usage. In-home greywater reuse is not widely practiced, and is illegal to reuse within homes throughout the majority of North America (National Research Council of the National Academics 2016), where there are many circumpolar communities still lacking sustainable water and sanitation (Thomas et al. 2016; Daley et al. 2015).

Current available technologies are capable of effectively treating greywater to potable quality; however, the cost of such systems is high (Cobacho et al. 2012) and not necessary for uses such as toilet flushing and clothes washing. There is a need for a cost effective and robust greywater treatment system that can handle the variability of contaminant composition within greywater and produce safe, disinfected, non-potable water for household uses ranging from toilet flushing to laundry, and potentially semi-continuous recirculation of shower water. Additionally, there is a need for regulatory guidelines to be designed for in-home greywater reuse including identifying adequate performance surrogates for greywater treatment processes to assess required pathogen log-reductions in risk-based guidelines (e.g. Sharvelle et al. 2017). Depending on the end use of the treated greywater, necessary log_10_ reductions may range from 5 to 13 for viruses, 4 to 9 for *Cryptosporidium*, 3 to 8 for *Giardia*, and 3 to 8 for bacteria (Schoen et al. 2017). Given complexities/costs in undertaking controlled spiking studies (Zimmerman et al. 2016), here we present the potential for using endogenous *Staphylococcus* spp. (total staphylococci) as a greywater treatment performance surrogate, demonstrated for ultraviolet (UV) irradiation performance testing.

### Ultraviolet irradiation

Disinfection is an essential process in water treatment to remove/inactivate pathogenic organisms. UV inactivation is a commonly used disinfection treatment method in both wastewater and drinking water treatment, which directly damages the nucleic acids of microorganisms and inhibits future replication (Gross et al. 2015). UV is an attractive disinfection method and considered to be a more environmentally friendly disinfection technology than chemical disinfection (Winward et al. 2008). Additionally, UV is often preferred over chlorine because the use of chlorine may leave residual chlorine compounds that may have adverse effects, such as generating odorous substances and biohazardous disinfection by-products (Chang et al. 1985; Mori et al. 2007). However, UV irradiation does have its limitations; Winward et al. (2008) showed raw greywater having higher turbidity, larger mean particle size, lower UV_254_ transmittance, and higher total suspended solids’ (TSS) levels relative to raw municipal wastewater. This can be explained by the lack of dilution of greywater when compared to municipal wastewater. Winward et al. (2008) also showed that the presence of larger particles in greywater limited the effectiveness of UV irradiation, causing an extreme tailing effect of coliforms reduction even in with UV doses up to 239 mJ cm^−2^. Hence, for UV irradiation to be an effective pathogen reduction step in greywater treatment, adequate pre-treatment must be performed to ensure adequate UV_254_ transmissivity and removal of larger particles capable of shielding microorganisms.

### Process indicators, faecal indicators and index organisms

There is often confusion between the semantics and purposes of process indicators (surrogates), faecal indicators, and pathogen index organisms. It is important to make a distinction between the use of indicators generally and surrogates specifically, as the roles of such are not necessarily interchangeable. Table 1 exhibits the key differences between the three.

**Table 1:** Definitions for indicator and index microorganisms of public health concern (World Health Organization 2001, 2016)

Group	Definition
Process indicator (surrogate)	A group or organism that demonstrates the efficacy of a process, such as total heterotrophic bacteria or total coliforms for chlorine disinfection.
Faecal indicator	A group or organism that indicates the presence of faecal contamination, such as the bacterial groups thermotolerant coliforms or *E. coli*. Hence, they only infer that pathogens may be present.
Index and model organisms	A group/or species indicative of pathogen presence and behaviour respectively, such as *E. coli* as an index for *Salmonella* presence and F-RNA coliphages as models of human enteric virus behaviour.

Process indicators (surrogates) and faecal indicator bacteria (FIB) are commonly used as indicators in water treatment to determine the potential presence of enteric viruses, bacteria and parasitic protozoan pathogens that maybe associated with faecal contamination (U.S. EPA 2012). The most commonly tested indicators are total coliforms, faecal coliforms, *Escherichia coli* (*E. coli*), and enterococci (U.S. EPA 2012). However, most greywater treatment studies inappropriately utilize FIB as an indicator of pathogen risk (Ottosson and Stenström 2003). Determining the microbiological contamination in greywater can be difficult; each source of greywater presents different potential biological contaminants and concentrations (Birks and Hills 2007). Zimmerman et al. (2014) investigated the 16S rRNA gene diversity of laundry water and identified skin-associated bacterial members of *Staphylococcus*, *Corynebacterium,* and *Propionibacterium* as the major members of that microbiome. Using quantitative polymerase chain reaction (qPCR) they showed that in the university gym laundry samples taken, *Staphylococcus* spp. averaged from 3 to 5 orders of magnitude higher than faecal source markers including total *Bacteroides* spp., human-specific *Bacteroides*, *Enterococcus* spp., and *E. coli*, in ascending orders of magnitude, respectively (Zimmerman et al. 2014). Due to the direct contact of clothing with human skin and the prevalence of the opportunistic pathogen *Staphylococcus aureus* (*S. aureus*) on the human body (Zimmerman et al. 2014), *S. aureus* may ﻿be at concentrations up to 5 × 10^5^ cfu.100 mL^−1^ (Burrows et al. 1991; Nolde 1999; Gilboa and Friedler 2008), but as with any pathogen, is not always present. Hence, *S. aureus*, enteric pathogens and FIB are present in varying and often low concentrations in greywater, with some of the FIB exhibiting growth within greywater systems (e.g. *E. coli*) (Ottosson and Stenström 2003). If regrowth occurs (either in the raw greywater holding tank or after treatment as reported by Friedler and Gilboa ( 2010), it is problematic to quantify the reduction occurring across any treatment step. Furthermore, traditional indicator organisms used in North America are not adequate surrogates to represent the log-reductions likely required to produce safe greywater (Sharvelle et al. 2017; Gilboa and Friedler 2008). Hence, the focus of this paper is to determine if endemic greywater staphylococci, including pathogens such as *S. aureus*, may be suitable treatment surrogates to reliably assay over 4-log_10_ reductions of key pathogens (Birks and Hills 2007; Zimmerman et al. 2014; Fogarty et al. 2015; Gross et al. 2007) by UV disinfection.

### Ultraviolet irradiation of staphylococci

Commercial UV systems generally deliver 254 nm UV-C from mercury-vapour lamps that impact on nucleic acid within microorganisms; however some manufacturers also use polychromatic UV-C lamps (including higher wavelength UV-C) that also impact on cellular proteins (Eischeid and Linden 2011). Little is known as to the efficacy of UV inactivation towards staphylococci, and in particular the pathogenic species *S. aureus* (Benami et al. 2013). Overall, there is a collective need for action-spectra and dose-response curves describing UV inactivation of *S. aureus*, as well as other *Staphylococcus* spp. generally and within greywater. Furthermore, given the above listed limitations with FIBs as process indicators for the removal of pathogens in greywater reuse, knowledge on the efficacy of UV inactivation towards staphylococci may provide future options for risk management monitoring. Most limiting is a lack of knowledge on the fate of pathogens (both enteric and saprozoic) via different greywater treatment systems, (Ashbolt 2015) and if demonstrated of value for enteric bacteria performance, total staphylococci may also provide value to address these additional pathogen concerns.

## Materials and methods

### Bacterial culturing, plating and enumeration

Freeze dried *S. aureus* (ATCC 25923), *Staphylococcus epidermidis* (*S. epidermidis*) (ATCC 12228), *Escherichia coli* (*E. coli*) (ATCC 13115), E. coli* (ATCC 15597), *Enterococcus faecalis* (*E. faecalis*) (ATCC 29212), and *Enterococcus casseliflavus* (*E. casseliflavus*) (ATCC 9199) were purchased from American Type Culture Collection (ATCC) and revived according to ATCC protocols. ATCC bacteria culture stocks were stored at −80 °C in a cryomedium to glycerol ratio of 3:2. When needed, bacteria cultures were inoculated into the brain hearth infusion (BHI) liquid media and incubated at 37 °C.

### Phage culturing, plating and enumeration

Aliquots (200 μL) of the F-RNA coliphages MS2 (ATCC 15597-B1) were used to infect E. coli* while in its exponential phase during incubation as described in Method 1601 (U.S. EPA 2001). Briefly, after 18 h of growth and infection time, the sample was filtered through a 0.22 μm filter to recover the coliphages and plaque within a semi-soft tryptic soy agar (TSA) overlay containing 0.7% agar. Plates were then incubated at 37 °C for 24 h before counting plaque-forming units (PFUs).

### UV irradiation

Liquid medium containing the test organism was diluted to 1:100 (for bacteria) and 1:1000 (for MS2 coliphage) in sterile deionized (DI) water inside a 60 mm Petri plate containing a 5 mm × 2 mm stir bar which was then placed on a magnetic stirrer at 400 rpm to facilitate mixing without a vortex forming. DI water was used rather than tap water in order to minimize potential contact with residual chlorine, which could have an inactivation effect on the cells (Zyara et al. 2016). Controls with bacteria suspended in sterile DI water were performed to investigate any loss in viability due to osmotic pressure change. No significant difference in log_10_ CFU count was observed from 100 s after pipetting from the BHI liquid media (the time needed to dilute the cells to a countable dilution) to 20 min (the estimated maximum time cells might be suspended in DI water throughout the experiment). Prior to exposing the sample to the UV, a 100-μL sample was pipetted out of the mixed solution and diluted to the appropriate dilution before being plated in triplicate. The collimated beam was then placed over the sample and the desired wavelength dose was delivered to the sample.

Based on an initial experiment performed for each test organism to determine the approximate dose needed for a 4-log_10_ reduction, a second experiment was performed to estimate an 11-point dose-response curve. Samples were taken at 0, 15, 30, 45, 60, 75, 90, 105, 120, 135, and 150% of the dose calculated for an estimated 4-log_10_ reduction. Subsamples (100 μL) were taken at appropriate times, diluted in DI water, and plated to determine the log_10_ reduction at each dosage using the following equation:

1 $$ {\mathrm{Log}}_{10}\kern0.2em  R e d u c t i o n= L o g\left(\frac{\frac{CFU\kern0.2em  Control}{100 mL}}{\frac{CFU\kern0.2em  Dosage}{100 mL}}\right) $$

An AquaSense Pearl Beam collimated UV reactor (Florence, KY USA) was used to deliver 255, 265, or 285 nm UV-C irradiation to test organisms suspended in water using a modified EPA protocol (U.S. EPA 2006). Equation 2 was used to calculate the effective intensity (*E*_ave_) of the collimated beam based on measurable variables: (NSF International 2014)

2 $$ {E}_{ave}=0.98\left[\frac{E_0}{L}\left(\frac{{\left(1- A\right)}^L-1}{\mathit{\ln}\left(1- A\right)}\right)\right] $$

The incident intensity (*E*_0_) was measured using a NSF certified radiometer calibrated for the three test wavelengths (UVP radiometer, model UVX-25). The water height (*L*) was measured to 1 cm (28.3 mL in a 60-mm cylindrical Petri dish), and a spectrophometer (Thermo Scientific GENESYS 10S UV-VIS) was used to measure the absorbance (A) of 255 nm UV by the suspending medium. The resulting *E*_ave_ was then multiplied by the exposure time (seconds) in order to calculate the resulting dosage measured in mJ cm^−2^.

## Results

**Fig. 1:**
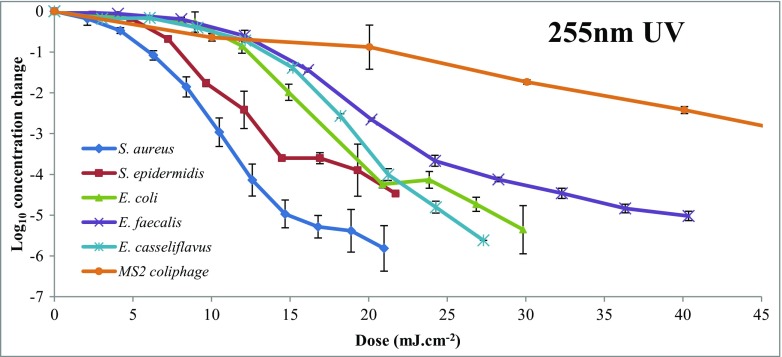
Comparison of inactivation of *Staphylococcus* spp., FIB and MS2 coliphage by 255 nm UV (average log reduction values ± SD)

Data points from each experiment were plotted to display the dose-response of each organism relative to 255 nm UV irradiation. Figure 1 shows the relative dose-response to UV by *S. aureus*, *S. epidermidis*, *E. faecalis*, *E. casseliflavus*, *E. coli,* and MS2 coliphage.

**Fig. 2:**
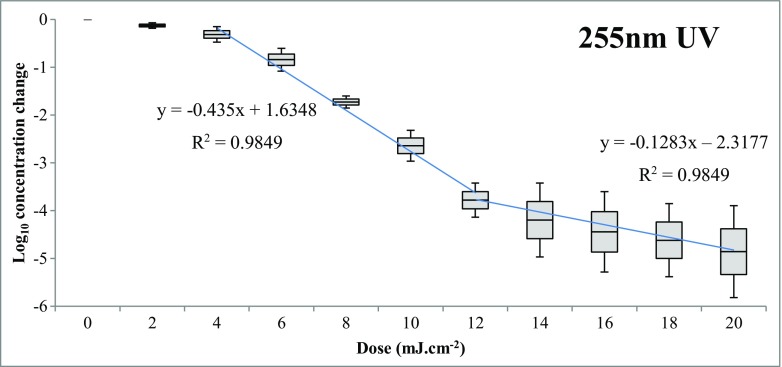
Bi-linear box and whisker plot for UV (255 nm) decay curve of total staphylococci (average of *S. aureus* and *S. epidermidis*)

*S. aureus* and *S. epidermidis* are generally the most common *Staphylococcus* spp. colonizing humans, (Coates et al. 2014) and therefore were used in order to represent total staphylococci in the bench experiments to determine the efficacy of UV inactivation. Data points for the *S. epidermidis* decay curve were interpolated to estimate log_10_ reduction values for the dosages for each *S. aureus* point. A weighted average was then taken of each point to estimate a dose-response curve for *Staphylococcus* spp., which is shown in Fig. 2. Figure 2 also depicts the bi-linear decay curve with total staphylococci; two trend lines were plotted to determine the decay equation (in units of Log_10_ Reduction (LR) per mJ cm^−2^) in the bi-linear graph to determine the decay coefficients K_1_ (0.435 LR per mJ cm^−2^) and K_2_ (0.1283 LR per mJ cm^−2^). These equations, along with the decay equations and respective *R* ^2^ values for the other test organisms are shown in Table 2. These equations were used to determine the relative reduction for each organism compared to total staphylococci.

**Table 2:** 255 nm UV-C decay curves for various organisms (linear equations represent the line of best fit for the linear segments of each decay curve)

Organism	Linear equation (x units: [mJ cm^−2^])	*R* ^2^
*Staphylococcus* spp.	y = −0.435x + 1.6348 (x < 12) y = −0.1283x − 2.3177 (12 < x < 20)	0.9849 0.973
*E. coli*	y = −0.343x + 3.0246 (9 < x < 21) y = −0.132x + 1.2697 (21 < 30)	0.9873 0.8306
*E. faecalis*	y = −0.258x + 2.5954 (12 < x < 24) y = −0.0847x − 1.6868 (24 < x < 40)	0.9956 0.9818
*E. casseliflavus*	y = −0.3527x + 3.8164 (14 < x < 25)	0.9849
MS2 coliphage	y = −0.0607x + 0.0323 (0 < x < 90)	0.9916

### Comparison of 255, 265, and 285 nm UV on *S. aureus* inactivation

Also explored was a comparative evaluation of 255, 265, and 285 nm UV irradiation on *S. aureus* to determine the most effective wavelength for inactivation. The initial experiment for 285 nm yielded very little inactivation and was not explored any further. The 255 nm (11.8 mJ cm^−2^ for 4-log_10_ reduction) UV wavelength was observed to be more effective in inactivating *S. aureus* than 265 nm (17.1 mJ cm^−2^ for a 4-log_10_ reduction).

## Discussion

Previously published data on FIB identified them as poor performance surrogates for greywater treatment (Ottosson and Stenström 2003; Birks and Hills 2007; Zimmerman et al. 2014). Key criteria for a suitable performance surrogate are given in Table 3, which compares FIB and *Staphylococcus* spp. Traditional FIB failed to satisfy the first three criteria, due to their inconsistent presence with varying, and generally low concentration (Ottosson and Stenström 2003; Zimmerman et al. 2014) and lack of correlation with pathogen presence; (Birks and Hills 2007) this is enough to determine that traditional FIB are not suitable performance surrogates for grey-water treatment. In contrast, total staphylococci meet the first three criteria due to their consistent and high concentration in greywater; (Zimmerman et al. 2014; Burrows et al. 1991; Nolde 1999; Gilboa and Friedler 2008) presumably, due to the high presence of staphylococci colonizing human skin (Coates et al. 2014) and the correlation of presence when human mitochondrial DNA (HmtDNA) is detected in greywater (Zimmerman et al. 2014). It is important to acknowledge that although some studies showed a non-detect of *S. aureus* in raw greywater samples (Casanova et al. 2001; Siegrist 1977) this is no surprise for a pathogen (as we are not always infected); whereas, other skin staphylococci are likely to be present. For example, Zimmerman et al. (2014) reported *S. aureus* at approximately 5-log_10_ lower concentration than *Staphylococcus* spp. in laundry greywater. In addition, approximately 30–40% of humans carry *S. aureus* (Kluytmans et al. 1997; Cole et al. 2001), while *Staphylococcus* spp. has been shown to dominate the microbiota of approximately 60% of humans (Callewaert et al. 2013). The fourth criteria explored, stating that an appropriate performance surrogate must have greater survival than target pathogens is still in question. More research is needed to determine the spectrum of genera present within a variety of greywater sources, and their treatment requirements for a 3.5 to 6-log_10_ removal, as likely needed for different domestic greywater reuse (Sharvelle et al. 2017). Although *Staphylococcus* spp. showed less resilience to UV than the FIB bacteria tested in the collimated UV beam bench test, total staphylococci are expected to be a minimum of 3-log_10_ higher in concentration than the FIB traditionally used. Due to the high and consistent concentrations of *Staphylococcus* spp., we are suggesting inactivation curves for each reference pathogen can be used to determine log_10_ reductions relative to *Staphylococcus* spp. (World Health Organization 2016).

**Table 3:** Comparison of FIB and *Staphylococcus* spp.

Criteria	FIB	*Staphylococcus* spp.
Consistent presence	✖	✓
Higher concentration than target pathogens	✖	✓
Identifiable correlation to presence of target pathogens	✖	✓
Same or greater survival as target pathogens	?	?

Since total staphylococci were observed to be more susceptible to inactivation by UV irradiation than FIB, *Staphylococcus* spp. inactivation cannot be used as enteric pathogen surrogates, such as *E. coli*, enterococci, and MS2 coliphage. In order to gauge the relative log_10_ reduction between S*taphylococcus* spp. and the other organisms which were tested, the linear equations for each organism was used to estimate the log_10_ reduction that would be observed relative to the dosage required for a 4 and 5-log_10_ reduction for *Staphylococcus* spp., respectively: being 13.0 and 20.9 mJ cm^−2^, respectively. These dosages were then used in the linear equations obtained from each decay curve (displayed in Table 2) to determine their relative log_10_ reduction to *Staphylococcus* spp. (Table 4).

**Table 4:** Log_10_ reduction credits relative to 4 and 5-log_10_ reduction of *Staphylococcus* spp.

Organism	Log_10_ reduction observed at 13.0 mJ cm^−2^	Log_10_ reduction observed at 20.9 mJ cm^−2^
*Staphylococcus* spp.	4	5
*E. coli*	1.4	4.1
*E. faecalis*	0.8	2.8
*E. casseliflavus*	0.8	3.6
MS2 coliphage	0.8	1.2

Given the high concentrations of *Staphylococcus* spp. present in greywater, relative to FIB (ranging from 3 to 5-log_10_ higher) (Zimmerman et al. 2014), total staphylococci may still serve as a conservative measure for pathogen reduction for enteric bacteria, especially at the dosage for an observed 5-log_10_ reduction of *Staphylococcus* spp.; the log_10_ reduction difference at 20.9 mJ cm^−2^ observed between *Staphylococcus* spp. and the enteric bacteria tested is less than 3-log_10_, which is satisfactory given the 3 to 5-log_10_ concentration difference reported by Zimmerman et al. (2014). However, the UV dosage required to achieve a 5-log_10_ reduction of *Staphylococcus* spp. is estimated to only achieve some 1.2-log_10_ reduction in MS2 coliphage; this suggests *Staphylococcus* spp. would likely not be an adequate surrogate for enteric virus reduction in greywater, and an additional surrogate, such as endogenous (staphylococci) bacteriophages, is needed to represent enteric virus reduction.
